# Metabolomic Profiles Associated with Obesity and Periodontitis during Pregnancy: Cross-Sectional Study with Proton Nuclear Magnetic Resonance (^1^H-NMR)-Based Analysis

**DOI:** 10.3390/metabo12111029

**Published:** 2022-10-27

**Authors:** Gerson Aparecido Foratori-Junior, Adrien Le Guennec, Tatiana Kelly da Silva Fidalgo, Leanne Cleaver, Marília Afonso Rabelo Buzalaf, Guy Howard Carpenter, Silvia Helena de Carvalho Sales-Peres

**Affiliations:** 1 Department of Pediatric Dentistry, Orthodontics and Public Health, Bauru School of Dentistry, University of São Paulo, Bauru 17012-901, Brazil; 2 Centre for Host-Microbiome Interactions, Faculty of Dental, Oral & Craniofacial Sciences, Guy’s Campus, King’s College London, London SE1 1UL, UK; 3 Nuclear Magnetic Resonance Facility, Guy’s Campus, King’s College London, London SE1 1UL, UK; 4 Department of Preventive and Community Dentistry, School of Dentistry, Rio de Janeiro State University, Rio de Janeiro 20551-030, Brazil; 5 Department of Biological Sciences, Bauru School of Dentistry, University of São Paulo, Bauru 17012-901, Brazil

**Keywords:** obesity, periodontitis, pregnancy, metabolomics, saliva

## Abstract

This study aimed to elucidate the metabolomic signature associated with obesity and periodontitis during pregnancy in plasma and saliva biofluids. Ninety-eight pregnant women were divided into: with obesity and periodontitis (OP = 20), with obesity but without periodontitis (OWP = 27), with normal BMI but with periodontitis (NP = 21), with normal BMI and without periodontitis (NWP = 30). Saliva and plasma were analyzed by ^1^H-NMR for metabolites identification. Partial Least Squares-Discriminant Analysis (PLS-DA), Sparse PLS-DA (sPLS-DA), and Variable Importance of Projection (VIP) were performed. ANOVA and Pearson’s correlation were applied (*p* < 0.05). Plasmatic analysis indicated the levels of glucose (*p* = 0.041) and phenylalanine (*p* = 0.015) were positively correlated with periodontal parameters and BMI, respectively. In saliva, periodontitis was mainly associated with high levels of acetic acid (*p* = 0.024), isovaleric acid, butyric acid, leucine, valine, isoleucine, and propionic acid (*p* < 0.001). High salivary concentrations of glycine (*p* = 0.015), succinic acid (*p* = 0.015), and lactate (*p* = 0.026) were associated with obesity. Saliva demonstrated a more elucidative difference than plasma, indicating the glucose-alanine cycle, alanine metabolism, valine, leucine and isoleucine degradation, glutamate metabolism, and Warburg effect as the main metabolic pathways.

## 1. Introduction

Obesity is a chronic and inflammatory disease that has been considered one of the major health problems around the world, since it may be related to other systemic conditions such as cardiovascular disease, diabetes mellitus, arterial hypertension, dyslipidemias, and cancers [1]. Worldwide, 39% of adults aged 18 years and over are diagnosed as overweight and 13% are diagnosed as obese. Previous epidemiological studies positively associated obesity with periodontal diseases [2,3]. A plausible biological model suggests that adipose tissue from individuals with obesity secretes inflammatory mediators which cause a generalized inflammatory state resulting in alveolar bone loss [1].

Periodontitis is a multifactorial, chronic, inflammatory disease that leads to loss of periodontal attachment to the tooth root surface and alveolar bone resorption [4]. Periodontitis is dependent on dysbiosis within the biofilm of human dental plaque [5], but there is an increase in the number of systemic diseases and conditions that are linked to periodontitis. Therefore, the extent and severity of tissue destruction appears to be influenced by host characteristics [6].

In the same way as obesity, pregnancy has been widely investigated and shown to be associated with periodontitis. Considering the high levels of gestational hormones, such as estrogen and progesterone, and reduced antimicrobial activity of peripheral neutrophils, pregnancy results in an altered immune response, and therefore increases inflammatory processes [7]. This mechanism associates pregnancy with acute periodontal inflammation. Recently, obesity and periodontitis were evaluated in pregnancy, showing a positive correlation [8]. Despite this, the literature investigating the pathophysiological mechanisms behind these associations is scarce.

In recent years, there has been an increase in studies of the human salivary metabolome. Studying the salivary metabolomic profile is elucidative since it can be used to understand metabolic pathways and as an easy instrument to identify and monitor early systemic and oral alterations. Untargeted metabolomics using mainly Nuclear Magnetic Resonance (NMR) spectroscopy and mass spectrometry have been previously adopted to evaluate the metabolites related to periodontitis [9,10,11,12,13,14,15,16,17,18,19,20,21,22,23,24,25]. In general, valine, phenylalanine, isoleucine, tyrosine, and butyrate were upregulated in periodontitis cases. However, there are no studies characterizing the metabolomic profile of pregnancy with periodontitis, or obesity in pregnancy with periodontal impairment. Therefore, we hypothesize that the concomitant conditions may affect a larger number of metabolic pathways. This study aimed to determine the metabolomic signature and perturbed metabolic pathways associated with obesity and periodontitis during pregnancy in saliva and plasma biofluids.

## 2. Materials and Methods

This observational, cross-sectional, and analytical study followed the Strengthening the Reporting of Observational Studies in Epidemiology (STROBE) guidelines [26] and was registered in the ReBEC (https://ensaiosclinicos.gov.br/rg/RBR-4jk8mk7) (accessed on 29 September 2022) under the protocol number RBR-4jk8mk7.

### 2.1. Ethical Approval Statement

In accordance with the Code of Ethics of the World Medical Association (Declaration of Helsinki, published in 1975 and revised in 2013), this study was approved by the Internal Ethics Committee from the Bauru School of Dentistry, University of São Paulo (CAAE 06624519.3.0000.5417; protocol code 3.284.822; approved on 17 April 2019). Individuals were included after written informed consent form.

### 2.2. Sampling Method

Participants were invited to participate if they fulfilled the following eligibility criteria: between 18–40 years old, in the 3rd trimester of pregnancy (27th–39th gestational week), having regular follow-up with an obstetrician; and who had adequate cognitive function during pregnancy, without impairments that required absolute rest. Participants were excluded if: this was a twin pregnancy, individuals had neuromotor impairments, arterial hypertension during pregnancy (blood pressure ≥ 140/90 mmHg), gestational diabetes mellitus (hyperglycemia: ≥ 92 mg/dL—fasting level; ≥180 mg/dL—after 1 h; and ≥153 mg/dL—after 2 h); malnutrition (BMI < 18.50 kg/m^2^), overweight (who were not obese nor eutrophic, so BMI between 25.00 kg/m^2^ and 29.99 kg/m^2^), confirmed or suspected diagnosis of SARS-CoV-2 infection, hyposalivation (<0.25 mL/min flow rate), they were taking antibiotics or had taken antibiotics during this pregnancy, were taking any medication that could interfere with periodontal status and/or salivary flow (e.g., immunosuppressive drugs, anticonvulsants or calcium channel blockers such as cyclosporine, phenytoin or nifedipine, respectively), they were under orthodontic treatment or any dental treatment with another professional, they had multiple tooth loss (more than two teeth per hemiarch), they had stage IV of periodontitis, self-reported systemic disease besides obesity, and users of alcohol/tobacco/illicit drugs.

**Figure 1:**
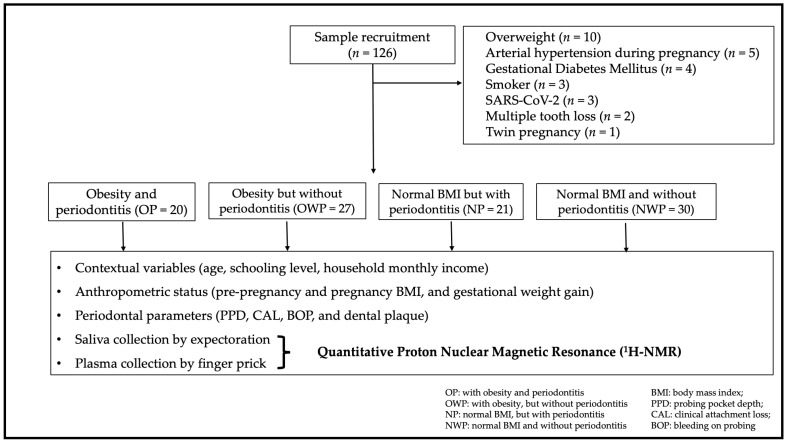
Sample recruitment and composition.

Participants were screened for inclusion from Primary Healthcare in Bauru, São Paulo, Brazil, between November 2020 and August 2021. Figure 1 shows the flowchart detailing the sample composition. All participants that were followed-up by health teams from Primary Healthcare units in Bauru during the period of recruitment, and who met our criteria, were included in this study. After recruitment, 98 pregnant women were divided into: with obesity and periodontitis (OP = 20); with obesity but without periodontitis (OWP = 27); with normal BMI but with periodontitis (NP = 21); with normal BMI and without periodontitis (NWP = 30). As this is a study addressing untargeted metabolomic analysis, sample size was based on previous evidence [12,16].

Pre-pregnancy BMI was deduced based on the World Health Organization (WHO), following previous studies [27,28,29,30,31,32,33]. Pregnant women with pre-pregnancy BMI ≥ 30.00 kg/m^2^ were allocated in the OP group or the OWP group, while those with BMI between 18.5–24.99 kg/m^2^ were allocated in the NP group or the NWP group. However, one patient with a borderline BMI of 18.3 kg/m^2^ was included in the NWP group. Pre-pregnancy weight was based on medical files from Primary Healthcare. Women’s height was obtained using a stadiometer (Wood 2.20; WCS Ind., Curitiba, Paraná, Brazil).

The periodontal parameters probing pocket depth (PPD) and clinical attachment level (CAL)/attachment loss (AL) were recorded by a previously calibrated dentist (kappa = 0.95 for clinical attachment level). Participants were diagnosed with periodontitis according to the classification described by Tonetti et al. (2018) [34]. Periodontitis cases were allocated to the OP or NP groups. Afterward, periodontitis cases were categorized in stages I, II, and III, according to the severity of the disease [34]. Considering the limitations (indications and contraindications) of the gestational period and respecting the ethical statements, the severity of the periodontitis was based only on the clinical parameters, and no dental radiographs were taken to avoid unnecessary exposure of pregnant women to X-rays.

### 2.3. Co-Variables

Age, schooling level, household monthly income, gestational weight gain, daily toothbrushing, daily flossing, prevalence of dental surfaces with visible plaque/biofilm [35] and of bleeding on probing (BOP) [36] were co-variables recorded. These co-variables were analyzed to ensure that the sample was homogeneous, minimizing the bias in the analysis of the metabolomic profile. The categorization of schooling level and household monthly income were described elsewhere [27,28,29,30,31,32,33]. Women’s weight during pregnancy was obtained using an automatic scale (MIC model 300PP, Micheletti Ind., São Paulo, Brazil).

### 2.4. Collection and Storage of Saliva and Plasma Samples

Following previous protocols [37,38], samples were collected in the morning, at least 1 h after oral exposure to exogenous substances (eating, chewing gum, or oral hygiene) or exercise. Unstimulated whole-mouth saliva (WMS) was collected by expectoration into sterilized universal tubes, over a period of 5 min. Blood was collected into heparinized capillaries by lancing the finger with a sterile lancet following disinfection with isopropanol. All fluids were kept on ice during processing. Saliva samples were centrifuged at 15,000× *g* for 10 min at 4 °C. The supernatant was collected. Blood samples were centrifuged at 1600× *g* for 15 min at 4 °C to yield plasma as described by Dona et al. [39]. Samples were stored at −80 °C prior to analysis.

### 2.5. Sample Preparation

A 540 µL aliquot of each saliva sample was mixed with 60 µL of NMR buffer in 5 mm outside diameter (OD) NMR tubes. NMR buffer for saliva was prepared as follow: 3 parts (V/V) deuterium oxide (99.9 atom % D, to provide a field frequency lock) (Sigma-Aldrich Corp. Milwaukee, WI, USA), and 1 part (V/V) stock of 2 mM of trimethylsilylpropanoic acid (TSP). The pH was adjusted to 7.4. Due to the low flow of plasma by finger prick collection, 90 µL of each plasma sample was mixed with 90 µL of NMR buffer in 3 mm OD NMR tubes. NMR buffer for plasma was prepared as follows: 1 part (V/V) deuterium oxide (99.9 atom % D, to provide a field frequency lock) (Sigma-Aldrich Corp. Milwaukee, WI, USA), 2 parts (V/V) of ultrapure water, and 1 part (V/V) stock of 2 mM of TSP. The pH was also adjusted to 7.4. In addition to individual analysis of the samples, a pooled saliva sample (15 µL of each saliva sample) and pooled plasma sample (4 µL of each plasma sample) were analyzed to ensure unambiguous assignment and as an external standard to verify the stability of the automation in the NMR analysis.

### 2.6. ^1^H-NMR Spectroscopy Analysis

Spectral acquisition and spectral processing are described in detail elsewhere [37,38]. All spectra were acquired on a Bruker Avance NEO 600 MHz equipped with a TCI Cryoprobe Prodigy (Bruker Biospin, Karlsruhe, Germany), operating at a proton frequency of 600.2 MHz at 298 K. For saliva samples, ^1^H spectra were acquired using Carr-Purcell-Meiboom-Gill (CPMG) pulse sequence with water suppression by presaturation [40], with 32 scans, 65,536 points for the free induction decay (FID) during the acquisition, a spectra width of 20.8 ppm, an acquisition time of 2.62 s, and an inter-scan delay of 4 s. Pooled samples were submitted to ^1^H-^1^H total correlation spectroscopy (TOCSY) experiment with water suppression by presaturation [40], 8 scans, 256 t1 increments, an echo time of 78.8 ms, a spectral width of 13.7 ppm in both dimensions, and a relaxation delay of 2 s; and using a ^1^H-^13^C heteronuclear single-quantum correlation (HSQC) experiment, with 16 scans, 256 t1 increments, a spectral width of 170 ppm in the ^13^C dimension and 12 ppm in the ^1^H dimension, and a relaxation time of 2 s. For plasma samples, CPMG pulse sequence and same parameters were also used with 64 scans. Samples were analyzed after a single freeze thaw cycle and were maintained at 4 °C prior to analysis. TSP peak (0 ppm) was used as internal reference/standard. As TSP can bind with certain plasma metabolites, the chemical shift peak from the CH_3_ of alanine (at 1.48 ppm) was used as an internal reference instead of the TSP peak in the plasma analysis.

Spectra were analyzed in TopSpin (version 4.0.3; Bruker Biospin, Karlsruhe, Germany). A 0.3 Hz exponential line broadening function was applied before Fourier transformation and automatic phase correction. Baselines were inspected and polynomial baseline correction applied. After processing the spectra on TopSpin, the spectra were exported into MATLAB software (version 2018b; MathWorks, Natick, MA, USA), where post-processing (alignment and normalization) was handled. The water region (4.2–5.1 ppm for saliva samples) and TSP peak were excluded before normalization. Peak alignment by fast Fourier transform (PAFFT method) and normalization by probabilistic quotient normalization (PQN) [41] were performed using a custom toolbox written by Clendinen et al. [42]. Metabolite identification was performed at the same time, and from that identification, regions that contained at least a fraction of a multiplet isolated for each metabolite was selected along with a few structures seen on 2D spectra but could not be assigned to a metabolite. The assignment was performed based on the human metabolome database (HMDB, http://www.hmdb.ca) (accessed on 13 July 2022). The resulting list of peak areas for each metabolite was converted to a .csv file to be exported to MetaboAnalyst 5.0 software (www.metaboanalyst.ca) (accessed on 13 July 2022), which was used for data analysis. Pareto scaling was also applied on MetaboAnalyst for normalization. 

### 2.7. Statistical Analysis and Bioinformatics

For clinical parameters, the Kolmogorov-Smirnov normality test was applied to analyze distribution of the data. Repeated measures of ANOVA with a post-hoc test (Scheffé) was applied for quantitative variables with normal distribution (age, gestational weight gain, and prevalence of dental plaque); Kruskal-Wallis with a post-hoc test (Dunn) was applied for quantitative variables without normal distribution (maternal BMI, daily toothbrushing, daily flossing, PPD, CAL, and prevalence of bleeding on probing).

For metabolomic analysis, PLS-DA and sPLS-DA were performed to obtain the predictive performance of the models; each model was evaluated for Q^2^, R^2^, and accuracy (ACC). For each model, 1000 permutations were performed. The variable importance in projection (VIP) scores was obtained based on PLS-DA for the determination of the relative abundances of the metabolites that contributed to the separation between the groups. Univariate analysis was performed through one-way ANOVA to evaluate whether the overall comparison was significant (post-hoc: Fisher’s least significant difference method—Fisher’s LSD). Correlations between metabolite expression and clinical parameters were also performed (Pearson’s correlation).

Metabolite set enrichment analysis (MSEA) was conducted to identify patterns of metabolite concentration changes considering the metabolite pathway and sub-class of metabolites [43]. Thus, we described the MSEA for visualization and functional analysis of metabolite data. Metabolites that presented differences in VIP score, loading, and univariate analysis for plasma (fatty acid, acetic acid, 3-hydroxybutirate, glucose, acetoacetic acid, formate, proline, isoleucine, alanine, citric acid, glycine, phenylalanine, choline, ethanol, histidine, asparagine, phenylalanine, leucine, ornithine, creatine phosphate, lactate, 3-hydroxybutyric acid, acetone, ornithine, and glucose) and saliva (acetic acid, propionic acid, ethanol, *N*-acetylglutamine, maltose, lactate, formic acid, glycine, 2,3-butanediol, glucose, succinic acid, alanine, pyruvic acid, butyric acid, choline, isovaleric acid, trimethylamine, leucine, valine, 2-methyl-3-ketovaleric acid, isoleucine, and 3-methyl-2-oxovaleric acid) were included.

## 3. Results

### 3.1. Characteristics of the Study Population

Groups did not differ regarding age (*p* = 0.082), schooling level (*p* = 0.155), and household monthly income (*p* = 0.292). Most individuals in all groups indicated that they had complete (51%) or incomplete (19.3%) secondary education. Regarding household monthly income, most of the participants indicated received up to 1 minimum wage (MW, approximately $240.00) (16.3%); up to 2 MW (36.7%) or up to 3 MW monthly (22.4%). The mean pre-pregnancy BMI was 32.87, 34.02, 22.43, and 23.05 kg/m^2^ for OP, OWP, NP, and NWP, respectively.

Groups did not differ regarding unstimulated salivary flow rate (0.74, 0.69, 0.71, and 0.68 mL/min for OP, OWP, NP, and NWP, respectively), daily toothbrushing, and daily flossing (*p* > 0.05). Dental plaque, BOP, PPD, and CAL were higher in the OP and NP groups (*p* < 0.001). Most individuals from the periodontitis groups were classified as stage II periodontitis (Table 1). 

### 3.2. Metabolomic Profiling of Plasma and Saliva

**Figure 2:**
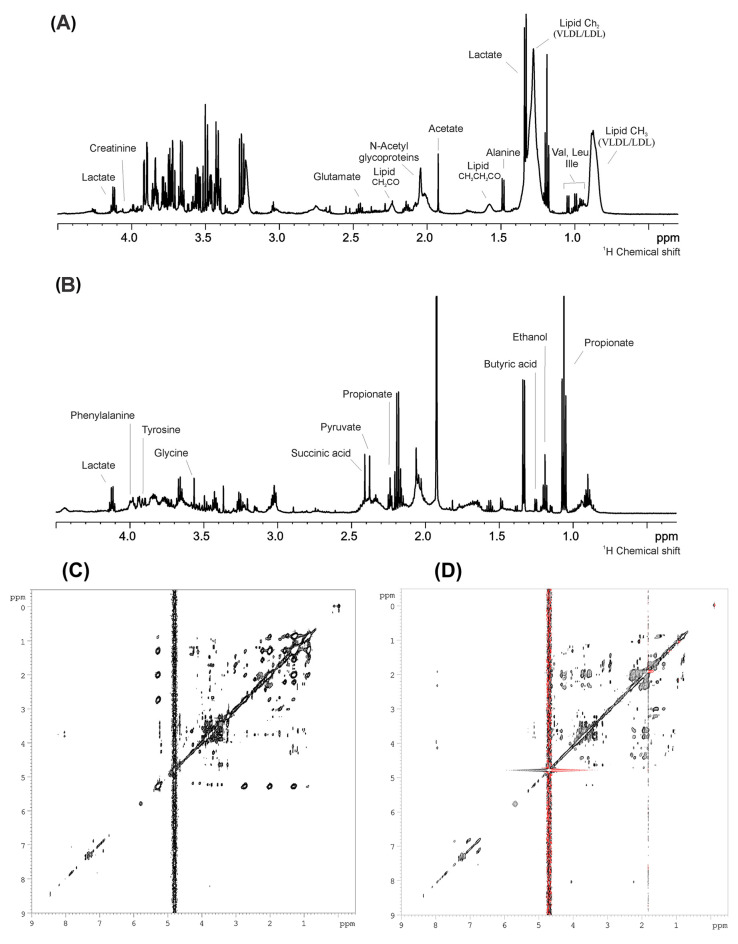
General view of 1D and 2D spectra of the pooled plasma ((**A**,**C**), respectively) and pooled saliva ((**B**,**D**), respectively) samples.

Figure 2 shows the general view of 1D and 2D spectra of the pooled plasma (A and C, respectively) and pooled saliva (B and D, respectively) samples.

**Figure 3:**
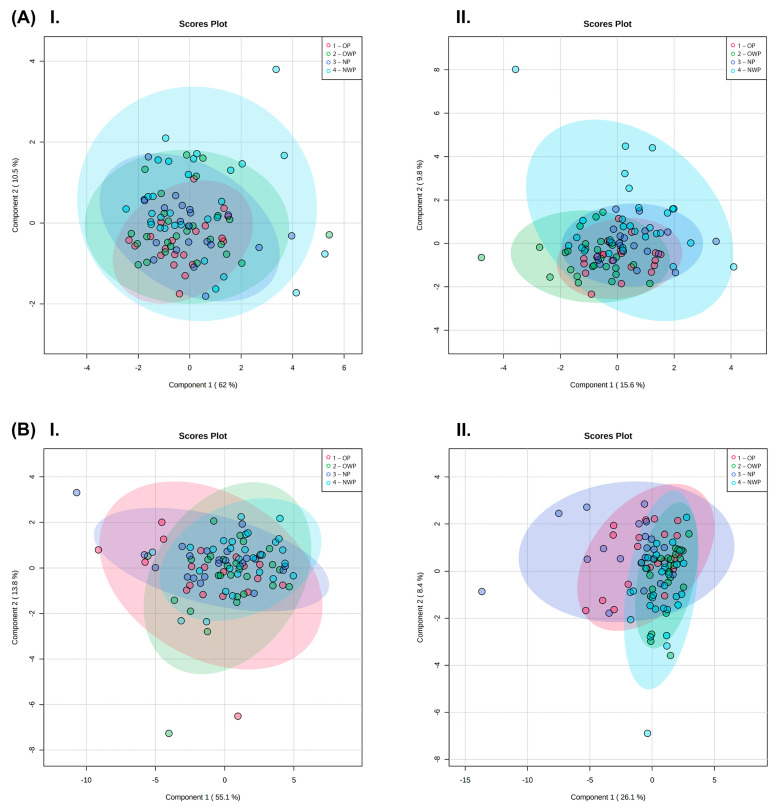
Plasma (**A**) and saliva (**B**) samples. Scores plot between the 1st and 2nd PCs identified by the PLS-DA (I); and scores plot between the 1st and 2nd PCs identified by the sPLS-DA (II).

Figure 3A shows the scores plot between the two first principal components (PCs) of PLS-DA (I) and sPLS-DA (II) of the plasma samples (ACC = 0.34; R^2^ = 0.35; Q^2^: −0.03). Figure 3B shows the scores plot between the 1st and 2nd PCs containing 68.9% of variation identified in the PLS-DA (I) and the scores plot between the 1st and 2nd PCs containing 34.5% variation in the sPLS-DA (II) of the saliva samples (ACC = 0.42; R^2^ = 0.25; Q^2^ = −0.75). Supplementary Figure S1 is related to hierarchical cluster analysis (I) and correlations (II) among the identified plasma (A) and saliva (B) metabolites.

**Figure 4:**
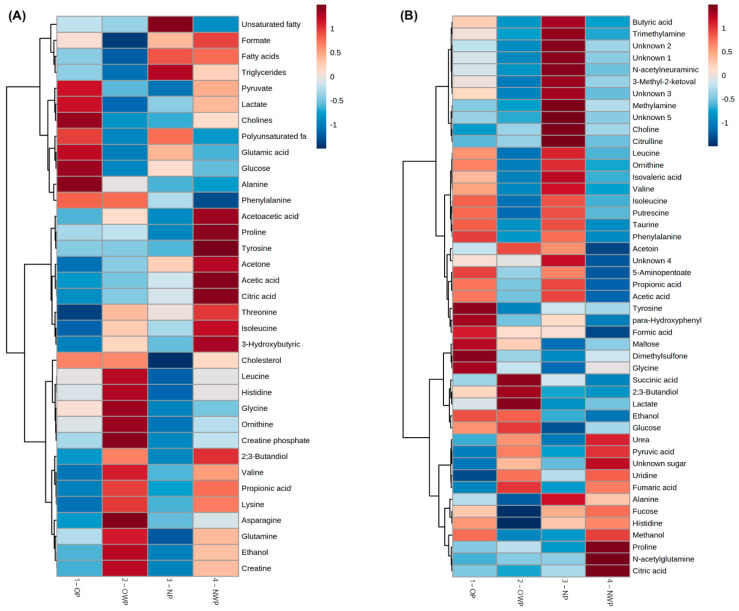
Heatmap with the groups average concentrations log-transformed of the identified plasma (**A**) and saliva (**B**) biofluids.

Figure 4 shows the heatmap of plasma (A) and saliva (B) metabolites, considering the average concentrations log-transformed. Analysis of metabolites from plasma showed that the OP group had higher levels of pyruvate, lactate, choline, polyunsaturated fatty acids, glutamic acid, glucose, and alanine. Phenylalanine and cholesterols were elevated in plasma samples of pregnant women with obesity (OP and OWP). Analysis of metabolites from saliva showed that phenylalanine, 5-aminopentoate, tyrosine, para-hydroxyphenylacetic acid, formic acid, maltose, dimethylsulfone, and glycine were increased in pregnant women with obesity and periodontitis. Ethanol and glucose were elevated in saliva samples of OP and OWP. Marked differences in salivary metabolites were demonstrated in participants with periodontitis, mainly in the NP group (i.e., higher levels of butyric acid, trimethylamine, *N*-acetylneuraminic, 3-methyl-ketovaleric acid, leucine, ornithine, isovaleric acid, valine, isoleucine, putrescine, taurine, propionic acid, and acetic acid).

Univariate analysis with ANOVA did not indicate significant differences among groups for plasma samples (Table 2), but important features were identified in the saliva samples (Table 3). Both Table 2 and Table 3 indicate the *p*-value related to the univariate analysis. However, even with a *p* > 0.05 for univariate analysis, if a particular metabolite also showed significance in the multivariate analysis (VIP or loading), this was indicated by superscript numbers in the second column of the tables after the name of the metabolites themselves.

Supplementary Table S1 shows the Pearson correlations among the identified plasma metabolites and clinical parameters (related to BMI and periodontal parameters). Leucine, isoleucine, acetoacetic acid, propionic acid, 3-hydroxybutyric, lysine, histidine, and cholesterol were negatively correlated with periodontal parameters (*p* < 0.05); while formate was negatively correlated with BMI (*p* = 0.033). In contrast, glucose was positively correlated with periodontal parameters (*p* < 0.05); while phenylalanine was positively correlated with BMI (*p* = 0.015).

Supplementary Table S2 shows the Pearson correlations among the identified salivary metabolites and clinical parameters related to BMI and periodontal parameters of pregnant women. Urea and uridine were negatively correlated with periodontal parameters (*p* < 0.001). In contrast, salivary 3-methyl-2-ketovaleric acid, butyric acid, isovaleric acid, leucine, valine, isoleucine, propionic acid, putrescine, acetic acid, *N*-acetylneuraminic acid, trimethylamine, ornithine, choline, taurine, phenylalanine, methylamine, alanine, and 5-aminopentoate were positively correlated with periodontal parameters (*p* < 0.05), while salivary 2,3-butanediol, succinic acid, glycine, lactate, and maltose were positively correlated with BMI (*p* < 0.05).

**Figure 5:**
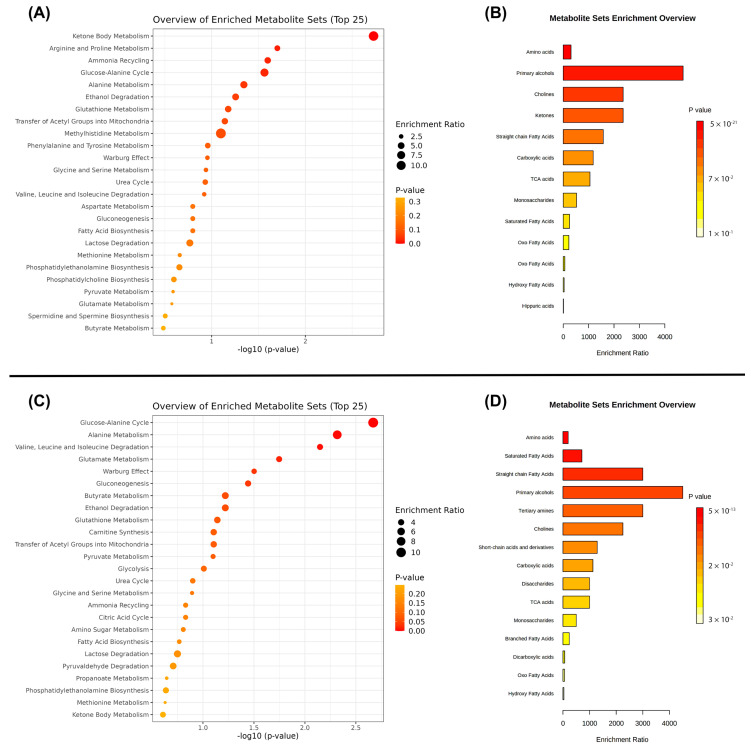
Main pathways and subclasses assigned by MSEA for plasma (**A**,**B**) and saliva (**C**,**D**) samples.

MSEA assigned the main metabolic pathways. For plasma, the five top pathways were: ketone body metabolism arginine and proline metabolism, ammonia recycling, glucose-alanine cycle, and alanine metabolism (Figure 5A). The main metabolic sub-classes were: amino acids, primary alcohols, cholines, ketones, and straight chain fatty acid (Figure 5B). For saliva, the top five pathways were: glucose-alanine cycle, alanine metabolism, valine, leucine and isoleucine degradation, glutamate metabolism, and Warburg effect (Figure 5C). The main metabolic subclasses were: amino acids, saturated fatty acids, straight chain fatty acids, primary alcohols, and tertiary amines (Figure 5D).

## 4. Discussion

This study sought to elucidate the metabolomic profile of plasma and saliva samples associated with obesity and periodontitis in pregnant women. Our results demonstrated a larger difference in metabolic activity in saliva samples compared with plasma, mainly related to periodontitis. Periodontal parameters were positively correlated with many saliva metabolites, showing significant changes in the levels of leucine, ornithine, isovaleric acid, valine, isoleucine, putrescine, taurine, phenylalanine, propionic acid, and acetic acid.

Periodontitis has been shown to be associated with both pregnancy and obesity [6]. The high levels of estrogen and progesterone during pregnancy, associated with a reduction in the antimicrobial activity of peripheral neutrophils, make women more susceptible to periodontal inflammation, even in the presence of a small amount of biofilm on the teeth [7,8]. Likewise, the adipose tissue of obese individuals secretes high levels of inflammatory mediators that cause a generalized inflammatory state of the body, making them susceptible to periodontal diseases [2,3]. There are limited publications in the literature that investigate the metabolic analysis of biofluids with a focus on understanding the pathophysiological mechanisms related to periodontitis and obesity during pregnancy.

Periodontal infections activate the host response to release many metabolic products at the periodontal pocket, and it is expected that salivary samples from patients with periodontitis show a specific metabolomic profile. Therefore, metabolic changes related to periodontitis may be a complementary method to evaluate the disease in addition to traditional clinical parameters, considering that some metabolites may even act as a potential biomarker of the disease.

In this study, salivary ornithine levels were positively correlated with periodontal parameters. We hypothesized that elevated ornithine levels in individuals with periodontal involvement is a physiological mechanism in the body to promote wound healing and tissue regeneration upon periodontal injuries. A previous animal experiment showed that ornithine is metabolized to form polyamines via ornithine decarboxylase and proline via ornithine aminotransferase. Polyamines contribute to cell proliferation and proline is essential in collagen synthesis. While both polyamines and proline are important for wound healing and tissue regeneration, they can also promote vascular fibrosis and thickening, resulting in arterial stiffening [44]. Considering that arterial stiffening is an important independent risk factor for cardiovascular diseases, increased levels of ornithine may be an important biomarker [44]. Nevertheless, we draw the attention to a higher risk of cardiovascular diseases in pregnant women with higher levels of this metabolite. The association between periodontal and cardiovascular diseases is already well characterized [45], but the causal correlation is still unclear; therefore, future studies should investigate the role of ornithine levels in the pathophysiology of these outcomes.

Previous evidence pointed out that lactate can be metabolized to acetate and propionate by *H. parainfluenzae*, *N. sicca*, *Eubacterium*, *Propionibacterium*, *Arachnia,* and *Veillonella* bacterial species [10,46]. We must consider the role of bacteria species in the oral metabolomic profile. The low concentration of lactate and higher levels of acetic acid and propionic acid in the saliva of individuals with the most severe periodontal parameters in this study (Figure 4B) may be due to changes in oral microbiome composition, which was not analyzed in this study. Microbiome analysis should be performed in future experiments to elucidate the role of these bacterial species in the metabolomic profile of saliva in pregnant women with periodontitis.

A number of amino acids were identified in this study. Valine, leucine, and isoleucine degradation were one of the main metabolic pathways highlighted here (Figure 5). Positive correlations were found between periodontal parameters and salivary concentrations of isoleucine, phenylalanine, and valine in this study as a consequence of the overall host tissue degradation, which in turn results in an ideal environment for bacterial proliferation and immune cell migration [10,16]. Plasma levels of phenylalanine were also significant here as they were positively correlated with individuals’ BMI. Previous evidence showed that high plasma levels of phenylalanine were a predictive factor for overweight/obesity [47]. In this study, saliva and plasma analysis facilitated the discovery of phenylalanine as a potential biomarker of the combination of obesity plus periodontitis during pregnancy. Future studies should further investigate the role of this metabolite in these outcomes.

This study demonstrated high salivary levels of valine (Figure 4B; Table 3) and low salivary concentrations of pyruvic acid (Figure 4B) in OP and NP groups. Valine can be synthetized by bacteria from pyruvic acid [48], which may explain our findings. Yet, there are two other mechanisms that justify the lower levels of pyruvic acid in periodontitis cases. Firstly, pyruvic acid represents the starting point of the citric acid cycle, which is upregulated in periodontal ligament cells during infection; and secondly, this metabolite is the principal substrate of *L*-lactate dehydrogenase, which exhibits increased activity during periodontal inflammation [48]. Furthermore, as periodontitis is a proteolytic disease, valine should be investigated in the future as an amino acid originating from the degradation of proteins by oral bacteria at the bottom of periodontal pockets.

The present study showed a positive correlation between salivary levels of taurine and periodontal parameters. The mobilization of taurine in the oral cavity may constitute a protective effect mediated by its antioxidant properties and gingival tissue regeneration, as this is known to be higher in inflammatory lesions [17,21]. Thus, taurine can protect tissues and maintain cellular homeostasis under inflammatory conditions and oxidative stress [21], modulating the inflammatory response within the periodontal tissues through, for instance, the production of pro-inflammatory cytokines such as *interleukins* (IL-1β, IL-6, and IL-8) [21].

Putrescine is a four-carbon diamine produced during tissue decomposition by the decarboxylation of amino acids, and it is directly related to cadaverine. In this study, putrescine was positively correlated with the periodontal parameters, agreeing with previous evidence in which elevated concentrations of cadaverine, as a consequence of protein degradation, were associated with higher periodontal pocket-inflamed surface area [48].

In this study, salivary levels of *N*-acetylneuraminic acid were also positively correlated with periodontal parameters. *N*-acetylneuraminic acid is the predominant sialic acid found in human cells and has been investigated as a potential biomarker of periodontitis [49]. Considering its potential as an inflammatory biomarker, sialic acid may have a regulatory role in immunological processes, preventing oxidative stress and removing reactive oxygen species [49]. Therefore, it is hypothesized that the *N*-acetylneuraminic was synthetized as an acute phase protein to limit injury and encourage healing in individuals with periodontitis in this study.

Many metabolic changes in glycolysis, the tricarboxylic acid cycle, and anaerobic respiration have been previously associated with periodontal diseases [48]. Previous analyses of metabolites related to energy production pathways had contradictory results [48]. Glucose was previously shown to increase in periodontitis cases [9,11], but in this study the findings related to glucose concentrations were controversial. Plasma glucose levels were positively correlated with periodontal parameters, while elevated salivary levels of glucose were more associated with individuals’ BMI (Figure 4B). Moreover, this study demonstrated the Warburg effect as an important pathway (Figure 5), indicating there is an increase in glucose uptake and preferential production of lactate [50]. Future studies in periodontitis cases must investigate the plasma and salivary profile in relation to glucose levels.

Salivary levels of ethanol and glycine were correlated with women’s BMI. A previous study revealed increased endogenous production of ethanol in the gastrointestinal tract of obese mice, which may result in hepatic steatosis [51]. Although studies report a reduction of glycine levels in individuals with obesity [52], recently untargeted metabolomics were combined with a bidirectional genetic approach to define the metabolome of obesity, and glycine was highlighted as one of the main metabolites to have more complex bidirectional cause-effect relationships with BMI [53]. In this study, pregnant women with obesity had higher levels of this metabolite when compared with women with normal BMI (Figure 4B). Glycine is a non-essential amino acid associated with obesity-related metabolic disorders, such as insulin resistance [54]. Considering the gestational period, we believe that this amino acid played an important role in carbon metabolism; therefore, its concentration imbalance was the result of the perturbation in cell proliferation and function [55].

In this study, high salivary levels of butyric acid and isovaleric acid were found to be associated with worse periodontal parameters. Most NMR-based metabolomic analyses showed that butyrate and isovalerate were upregulated in patients with periodontitis [21,48], and the increase in their levels is an important indicator of the growth of pathogenic subgingival microorganisms (such as *Porphyromonas*, *Prevotella,* and *Fusobacterium* species), and of the progression of periodontal tissue destruction [21,22]. The complex interplay between the host immune system and dysbiotic microflora is also reflected by changes, for instance, in the salivary levels of butyrate and succinate [48]. Recently, *Porphyromonas gingivalis*-infected periodontal ligament cells showed high levels of succinate [56], being directly associated with energetic stress. In this study, higher salivary levels of succinic acid were positively correlated with patients’ BMI, regardless of the periodontal status. We hypothesized that this occurred due to the enhanced secretion of pro-inflammatory lipids by inflammatory cells and by the upregulation of fatty acid metabolism in response to oxidative stress [48] related to obesity in those women from OP and OWP groups. Nevertheless, we emphasize that future studies investigating the role of bacteria, specifically, in the metabolomic profile of pregnant women with obesity and periodontitis are needed.

This study has some limitations that need to be highlighted. As pointed out by Romano and collaborators [16,18], metabolites defining the periodontal status mainly reflect bacterial species (lactate, pyruvate, formate) [13], tissue degradation (phenylalanine) [9], and host immune response (valine, isoleucine) [16]. Saliva contains several metabolites which may originate from heterogeneous sources (not only from the host, but also from diet and oral bacteria, from both supra and subgingival communities). In this study, only saliva samples from the host were evaluated for metabolomics, without any evaluation of the salivary bacterial profile. Yet, Kuboniwa et al. [12] proposed a supragingival scaling prior to sample collection to control the heterogeneity of the studied metabolites, but in this study a scaling session was not performed. Although Kim et al. [21] did not find any marked difference between stage II/stage III periodontitis groups and metabolomic profile, metabolites from subgingival pockets in saliva are more likely to be masked by irrelevant metabolites found in moderate cases of periodontitis [12]. Therefore, another limitation of this study was the fact that individuals diagnosed with stage I periodontitis were grouped with those diagnosed with stages II and III of the disease.Finally, the cross-sectional design of this study is considered another limitation. Ideally, to gain a cause-and-effect understanding of outcomes, women should be recruited before pregnancy and followed through the gestational trimesters, and also after delivery. Therefore, prospective studies with a larger sample size should be conducted in the future.

## 5. Conclusions

Saliva samples showed more expressive metabolomic changes than plasma samples, mainly associated with periodontitis regardless of BMI. The main metabolic pathways were the glucose-alanine cycle; alanine metabolism; valine, leucine, and isoleucine degradation; glutamate metabolism; and Warburg effect. In summary, periodontitis was associated with changes in the salivary levels of leucine, ornithine, isovaleric acid, valine, isoleucine, putrescine, taurine, phenylalanine, propionic acid, and acetic acid.

## Figures and Tables

**Table 1:** Systemic condition, oral hygiene behavior, and periodontal parameters of the sample.

Variables	OP (*n* = 20)	OWP (*n* = 27)	NP (*n* = 21)	NWP (*n* = 30)	*p*
	Mean ± SD	Mean ± SD	Mean ± SD	Mean ± SD	
	Median	Median	Median	Median	
	(1st–3rd Quartiles)	(1st–3rd Quartiles)	(1st–3rd Quartiles)	(1st–3rd Quartiles)	
Age (years)	29.85 ± 5.32	27.38 ± 6.05	25.33 ± 5.06	27.16 ± 5.80	0.082 *
Pre-pregnancy BMI (kg/m^2^)	31.43	32.51	23.30	23.27	**<0.001 ^†^**
	(30.09–35.04)	(30.30–36.51)	(20.43–24.80)	(22.26–24.65)	
	**A**	**A**	**B**	**B**	
Pregnancy BMI (kg/m^2^)	36.01	35.96	26.67	25.69	**<0.001 ^†^**
	(31.50–39.36)	(32.80–39.92)	(23.99–29.62)	(23.48–29.43)	
	**A**	**A**	**B**	**B**	
Gestational weight gain (kg)	7.25 ± 6.73	8.00 ± 5.60	10.38 ± 6.84	10.98 ± 5.29	0.095 *
Daily toothbrushing	3	3	3	3	0.585 ^†^
	(2–3)	(2–3)	(2–3)	(2–3)	
Daily flossing	0	1	0	0	0.073 ^†^
	(0–0)	(0–1)	(0–1)	(0–1)	
Dental plaque (%)	76.59 ± 20.56	55.74 ± 25.25	77.62 ± 18.19	62.71 ± 21.33	0.001 *
	**A**	**B**	**A**	**AB**	
BOP (%)	61.31	34.22	78.66	35.18	**<0.001 ^†^**
	(48.62–75.99)	(26.19–46.42)	(65.03–87.22)	(29.76–47.02)	
	**A**	**B**	**A**	**B**	
PPD (mm)	2.45	2.03	2.54	2.06	**< 0.001 ^†^**
	(2.23–2.64)	(1.98–2.10)	(2.42–2.78)	(2.00–2.16)	
	**A**	**B**	**A**	**B**	
CAL (mm)	2.45	2.06	2.54	2.08	**<0.001 ^†^**
	(2.25–2.65)	(2.02–2.11)	(2.43–2.80)	(2.01–2.16)	
	**A**	**B**	**A**	**B**	
Periodontitis stages (PS)–*n* (%)		-		-	-
I	11 (55%)		9 (42.9%)		
II	8 (40%)		10 (47.6%)		
III	1 (5%)		2 (9.5%)		
IV	0		0		

SD, standard deviation; *p*, significance level; BMI, body mass index; BOP, bleeding on probing; PPD, probing pocket depth; CAL, clinical attachment level; PS, periodontitis stages; * ANOVA (post-hoc test: Scheffé); ^†^ Kruskal-Wallis (post-hoc test: Dunn). Different bold letters mean statistically significant differences between groups. Bold values, statistical significance lower than 5%.

**Table 2:** Main plasma metabolite concentration shown as the mean, standard deviation, chemical shift (ppm), and statistical analysis from multivariate analysis using the (1) VIP scores, (2) loading factor and (3) univariate analysis.

HMDB Card	Metabolites	CS (ppm)	OP (*n* = 20) Mean ± SD	OWP (*n* = 27) Mean ± SD	NP (*n* = 21) Mean ± SD	NWP (*n* = 30) Mean ± SD	*p*
HMDB0000687	Leucine ^2^	0.96	0.41 ± 0.04	0.42 ± 0.05	0.39 ± 0.03	0.41 ± 0.05	0.192
HMDB0000172	Isoleucine ^1,3^	1.01	0.15 ± 0.01 **A**	0.16 ± 0.02 **AB**	0.16 ± 0.02 **AB**	0.17 ± 0.03 **B**	**0.047**
HMDB0000108	Ethanol ^1,2^	1.17	1.30 ± 0.83	2.45 ± 4.00	1.09 ± 0.51	1.89 ± 3.02	0.313
-	Fatty acids ^1^	1.17	32.47 ± 5.50	32.11 ± 8.34	33.05 ± 8.07	33.01 ± 9.97	0.973
HMDB0000011	3-Hydroxybutyric acid ^1^	1.20	0.47 ± 0.07	0.51 ± 0.14	0.48 ± 0.08	0.55 ± 0.23	0.267
HMDB0000161	Alanine ^1^	1.48	0.77 ± 0.09	0.74 ± 0.11	0.73 ± 0.11	0.73 ± 0.10	0.676
HMDB0000042	Acetic acid ^1^	1.92	0.66 ± 0.15	0.68 ± 0.14	0.69 ± 0.15	0.77 ± 0.27	0.223
HMDB0001659	Acetone ^1^	2.22	0.17 ± 0.04	0.18 ± 0.05	0.19 ± 0.06	0.20 ± 0.07	0.406
HMDB0000060	Acetoacetic acid ^1^	2.27	0.13 ± 0.03	0.14 ± 0.05	0.12 ± 0.02	0.16 ± 0.09	0.083
HMDB0000168	Asparagine ^2^	2.87	0.04 ± 0.00	0.05 ± 0.00	0.05 ± 0.00	0.05 ± 0.00	0.084
HMDB0000214	Ornithine ^2^	3.05	0.04 ± 0.00	0.04 ± 0.00	0.04 ± 0.00	0.04 ± 0.00	0.282
HMDB0000097	Choline ^1^	3.10	2.40 ± 0.19	2.33 ± 0.29	2.33 ± 0.31	2.36 ± 0.30	0.823
HMDB0000123	Glycine ^1,2^	3.56	0.35 ± 0.06	0.37 ± 0.07	0.33 ± 0.04	0.34 ± 0.07	0.231
HMDB0250525	Creatine phosphate ^2^	3.96	0.05 ± 0.00	0.06 ± 0.00	0.05 ± 0.00	0.05 ± 0.01	0.407
HMDB0000190	Lactate ^2^	4.11	0.84 ± 0.17	0.73 ± 0.17	0.76 ± 0.13	0.80 ± 0.19	0.127
HMDB0000162	Proline ^1^	4.15	0.09 ± 0.04	0.10 ± 0.04	0.08 ± 0.01	0.12 ± 0.09	0.127
HMDB0000122	Glucose ^1^	4.65	1.70 ± 0.41	1.57 ± 0.23	1.63 ± 0.26	1.59 ± 0.27	0.524
HMDB0000177	Histidine ^2^	7.08	0.05 ± 0.00	0.06 ± 0.01	0.05 ± 0.00	0.05 ± 0.00	0.053

HMDB, human metabolome database; CS, chemical shift, OP, obesity and periodontitis; OWP, obesity without periodontitis; NP, normal BMI with periodontitis; NWP, normal BMI without periodontitis; SD, standard deviation; *p*, significance level; ^1^ multivariate analysis using VIP scores; ^2^ multivariate analysis using the loading factor; ^3^ univariate analysis (ANOVA with post-hoc Fisher’s LSD). Different bold letters mean statistically significant differences between groups. Bold values, statistical significance lower than 5%.

**Table 3:** Main saliva metabolite concentration shown as the mean, standard deviation, chemical shift (ppm), and statistical analysis from multivariate analysis using the (1) VIP scores, (2) loading factor, and (3) univariate analysis.

HMDB Card	Metabolites	CS (ppm)	OP (*n* = 20) Mean ± SD	OWP (*n* = 27) Mean ± SD	NP (*n* = 21) Mean ± SD	NWP (*n* = 30) Mean ± SD	*p*
HMDB0000491	3-Methyl-2-ketovaleric acid ^2,3^	0.85	0.29 ± 0.08 **AB**	0.24 ± 0.04 **A**	0.34 ± 0.13 **B**	0.27 ± 0.05 **A**	**0.001**
HMDB0000039	Butyric acid ^1,2,3^	0.89	0.51 ± 0.19 **AB**	0.37 ± 0.14 **A**	0.65 ± 0.33 **B**	0.38 ± 0.16 **A**	**<0.001**
HMDB0000718	Isovaleric acid ^2,3^	0.91	0.31 ± 0.16 **A**	0.20 ± 0.07 **B**	0.38 ± 0.19 **A**	0.22 ± 0.07 **B**	**<0.001**
HMDB0000687	Leucine ^2,3^	0.97	0.10 ± 0.04 **A**	0.07 ± 0.01 **B**	0.10 ± 0.03 **A**	0.08 ± 0.02 **B**	**<0.001**
HMDB0000883	Valine ^2,3^	0.99	0.18 ± 0.09 **AB**	0.13 ± 0.05 **A**	0.20 ± 0.08 **B**	0.13 ± 0.05 **A**	**<0.001**
HMDB0000172	Isoleucine ^2,3^	1.01	0.10 ± 0.06 **A**	0.06 ± 0.02 **B**	0.10 ± 0.04 **A**	0.07 ± 0.03 **AB**	**0.008**
HMDB0000237	Propionic acid ^1,2,3^	1.06	6.03 ± 3.00 **AB**	4.79 ± 2.25 **AB**	6.23 ± 3.45 **A**	4.10 ± 2.33 **B**	**0.020**
HMDB0003156	2,3-Butanediol ^1^	1.14	0.31 ± 0.21	0.37 ± 0.47	0.26 ± 0.09	0.25 ± 0.25	0.444
HMDB0000108	Ethanol ^1^	1.18	1.51 ± 0.91	1.50 ± 1.05	1.28 ± 0.60	1.23 ± 0.59	0.489
HMDB0000161	Alanine ^1^	1.48	0.53 ± 0.16	0.49 ± 0.13	0.60 ± 0.24	0.56 ± 0.17	0.210
HMDB0000042	Acetic acid ^1^	1.92	21.08 ± 9.94	17.96 ± 6.90	21.65 ± 9.50	16.30 ± 6.65	0.071
HMDB0006029	*N*-Acetylglutamine ^1^	2.33	1.23 ± 0.32	1.25 ± 0.37	1.26 ± 0.55	1.48 ± 0.55	0.166
HMDB0000243	Pyruvic acid ^1^	2.37	1.10 ± 0.29	1.19 ± 0.36	1.11 ± 0.31	1.21 ± 0.36	0.610
HMDB0000254	Succinic acid ^1^	2.41	0.91 ± 0.40	1.08 ± 1.16	0.93 ± 0.66	0.86 ± 0.40	0.723
HMDB0000906	Trimethylamine ^2,3^	2.89	0.07 ± 0.05 **A**	0.05 ± 0.02 **A**	0.12 ± 0.09 **B**	0.05 ± 0.02 **A**	**<0.001**
HMDB0000097	Choline ^1,3^	3.20	0.26 ± 0.08 **A**	0.28 ± 0.10 **A**	0.36 ± 0.12 **B**	0.28 ± 0.06 **A**	**0.009**
HMDB0001875	Methanol ^3^	3.36	0.26 ± 0.25 **AB**	0.18 ± 0.07 **A**	0.19 ± 0.06 **A**	0.27 ± 0.12 **B**	**0.038**
HMDB0000123	Glycine ^1^	3.56	0.58 ± 0.54	0.46 ± 0.20	0.39 ± 0.16	0.47 ± 0.20	0.247
HMDB0000190	Lactate ^1^	4.12	1.23 ± 1.23	1.62 ± 1.94	1.03 ± 0.46	1.12 ± 0.59	0.344
HMDB0000122	Glucose ^1^	5.24	0.30 ± 0.65	0.33 ± 0.52	0.18 ± 0.22	0.24 ± 0.35	0.696
HMDB0000163	Maltose ^1^	5.41	0.41 ± 1.63	0.26 ± 0.67	0.05 ± 0.06	0.15 ± 0.32	0.542
HMDB0000159	Phenylalanine ^3^	7.43	0.09 ± 0.05 **AB**	0.06 ± 0.02 **A**	0.09 ± 0.03 **B**	0.06 ± 0.02 **A**	**0.027**

HMDB, human metabolome database; CS, chemical shift, OP, obesity and periodontitis; OWP, obesity without periodontitis; NP, normal BMI with periodontitis; NWP, normal BMI without periodontitis; SD, standard deviation; *p*, significance level; ^1^ multivariate analysis using VIP scores; ^2^ multivariate analysis using the loading factor; ^3^ univariate analysis (ANOVA with post-hoc Fisher’s LSD). Different bold letters mean statistically significant differences between groups. Bold values, statistical significance lower than 5%.

## Data Availability

The data presented in this study are available within this article and in the Supplementary Figure S1, and Supplementary Tables S1 and S2.

## Supplementary Materials

The following supporting information can be downloaded at: https://www.mdpi.com/article/10.3390/metabo12111029/s1, Supplementary Figure S1: Hierarchical cluster analysis (I) and correlations (II) among the identified plasma (A) and saliva (B) metabolites; Supplementary Table S1: Pearson correlation among plasmatic metabolites and co-variables related to BMI and periodontal parameters; Supplementary Table S2: Pearson correlation among the salivary metabolites and co-variables related to BMI and periodontal parameters.

## Abbreviations

^1^H-NMR: quantitative proton nuclear magnetic resonance; ACC: accuracy; AL: attachment loss; ANOVA: analysis of variance; BMI: body mass index; BOP: bleeding on probing; CAL: clinical attachment level; CPMG: Carr-Purcell-Meiboom-Gill; CS: chemical shift; FID: free induction decay; *H. parainfluenzae*: *Haemophilus parainfluenzae*; HMDB: human metabolome database; HSQC: heteronuclear single-quantum correlation; IL: interleukin; LSD: least significant difference; MSEA: metabolite set enrichment analysis; MW: minimum wage; *N. sicca*: *Neisseria sicca*; NMR: nuclear magnetic resonance; NP: normal BMI with periodontitis; NWP: normal BMI without periodontitis; OD: outside diameter; OP: obesity and periodontitis; OWP: obesity without periodontitis; P: significance level; PAFFT: peak alignment by fast Fourier transform; PCs: principal components; PLS-DA: partial least squares-discriminant analysis; PPD: probing pocket depth; PQN: probabilistic quotient normalization; PS: periodontitis stages; sPLS-DA: sparse partial least squares-discriminant analysis; STROBE: strengthening the reporting of observational studies in epidemiology; TOCSY: total correlation spectroscopy; TSP: trimethylsilylpropanoic acid; VIP: variable importance in projection; WMS: whole-mouth saliva.
