# Supplementary material for: Metabolomic Profiles Associated with Obesity and Periodontitis during Pregnancy: Cross-Sectional Study with Proton Nuclear Magnetic Resonance (1H-NMR)-Based Analysis
Source: Metabolites. 2022 Oct 27;12(11):1029. doi: 10.3390/metabo12111029 (PMC9694155; doi:10.3390/metabo12111029)
Supplement: Supplementary file 1 [file metabolites-12-01029-s001.zip › metabolites-1972087-supplementary.pdf]

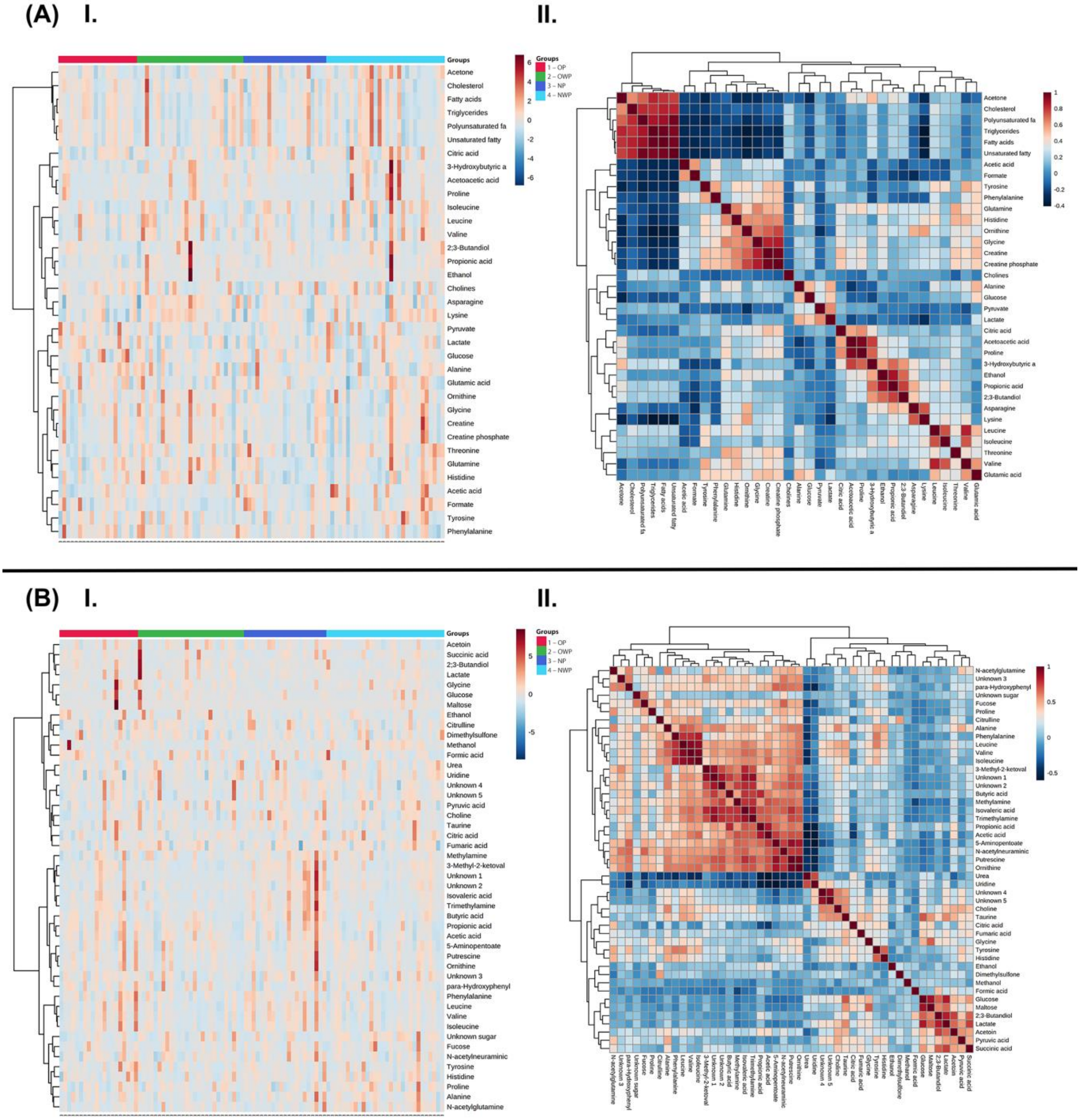

Figure S1. Hierarchical cluster analysis (I) and correlations (II) among the identified plasma (A) and saliva (B) metabolites.

Table S1. Pearson correlation among plasmatic metabolites and co-variables related to BMI and periodontal parameters.

Metabolites (chemical shift – ppm)		BMI	PPD	CAL	PS	Dental plaque	BOP
**Cholesterol (0.59)**	Pearson Correlation	0.002	**-0.208***	-0.191	-0.123	-0.078	0.062
	Sig. (2-tailed)	0.987	**0.040**	0.060	0.227	0.443	0.542
**Leucine (0.96)**	Pearson Correlation	0.116	**-0.205***	**-0.211***	**-0.204***	-0.039	0.040
	Sig. (2-tailed)	0.254	**0.042**	**0.037**	**0.044**	0.701	0.699
Valine (0.98)	Pearson Correlation	0.012	-0.145	-0.157	-0.167	0.003	0.001
	Sig. (2-tailed)	0.909	0.154	0.124	0.100	0.979	0.992
**Isoleucine (1.01)**	Pearson Correlation	-0.160	**-0.241***	**-0.255***	**-0.246***	-0.143	-0.100
	Sig. (2-tailed)	0.115	**0.017**	**0.011**	**0.015**	0.160	0.329
**Propionic acid (1.07)**	Pearson Correlation	-0.053	-0.185	-0.191	**-0.250***	-0.123	-0.128
	Sig. (2-tailed)	0.604	0.069	0.059	**0.013**	0.229	0.209
2,3-Butanediol (1.14)	Pearson Correlation	-0.016	-0.126	-0.129	-0.121	-0.013	-0.090
	Sig. (2-tailed)	0.874	0.217	0.206	0.234	0.895	0.377
Fatty acids (1.17)	Pearson Correlation	-0.114	-0.132	-0.114	-0.048	-0.090	0.108
	Sig. (2-tailed)	0.263	0.195	0.263	0.639	0.379	0.289
Ethanol (1.17)	Pearson Correlation	0.045	-0.125	-0.124	-0.163	-0.122	-0.121
	Sig. (2-tailed)	0.662	0.220	0.223	0.109	0.231	0.235
**3-Hydroxybutyric acid (1.20)**	Pearson Correlation	-0.096	-0.198	-0.186	**-0.207***	0.000	-0.158
	Sig. (2-tailed)	0.347	0.050	0.066	**0.041**	0.998	0.121
Alanine (1.48)	Pearson Correlation	0.016	0.029	0.003	0.009	0.023	0.126
	Sig. (2-tailed)	0.876	0.775	0.973	0.931	0.821	0.217
Acetic acid (1.92)	Pearson Correlation	-0.126	0.039	0.025	-0.057	-0.051	0.034
	Sig. (2-tailed)	0.215	0.706	0.808	0.574	0.619	0.743
Acetone (2.22)	Pearson Correlation	-0.181	-0.182	-0.171	-0.113	-0.119	-0.017
	Sig. (2-tailed)	0.075	0.073	0.093	0.270	0.242	0.867
**Acetoacetic acid (2.27)**	Pearson Correlation	-0.031	**-0.214***	**-0.202***	**-0.241***	0.025	-0.177
	Sig. (2-tailed)	0.764	**0.034**	**0.046**	**0.017**	0.809	0.080
Pyruvate (2.37)	Pearson Correlation	-0.027	-0.056	-0.068	-0.041	-0.039	-0.104
	Sig. (2-tailed)	0.791	0.587	0.505	0.686	0.705	0.310
Glutamic acid (2.34)	Pearson Correlation	-0.022	0.059	0.067	0.104	0.063	0.165
	Sig. (2-tailed)	0.827	0.565	0.512	0.309	0.539	0.104
Glutamine (2.45)	Pearson Correlation	0.178	-0.108	-0.106	-0.168	0.101	-0.072
	Sig. (2-tailed)	0.079	0.292	0.297	0.098	0.324	0.483
Citric acid (2.53)	Pearson Correlation	-0.171	-0.095	-0.064	-0.066	-0.039	-0.058
	Sig. (2-tailed)	0.092	0.353	0.531	0.516	0.702	0.573
Polyunsaturated fatty acids (2.63)	Pearson Correlation	-0.057	-0.110	-0.100	-0.034	-0.068	0.092
	Sig. (2-tailed)	0.578	0.280	0.329	0.740	0.508	0.370
Asparagine (2.87)	Pearson Correlation	0.092	-0.096	-0.104	-0.184	0.015	-0.090
	Sig. (2-tailed)	0.366	0.348	0.308	0.069	0.886	0.380
**Lysine (3.02)**	Pearson Correlation	-0.035	-0.147	-0.150	**-0.237***	0.019	-0.114
	Sig. (2-tailed)	0.735	0.149	0.141	**0.019**	0.850	0.265
Ornithine (3.05)	Pearson Correlation	0.112	-0.118	-0.084	-0.128	0.035	-0.098
	Sig. (2-tailed)	0.272	0.249	0.411	0.211	0.730	0.338
Choline (3.10)	Pearson Correlation	-0.048	0.050	0.051	-0.029	0.143	0.071
	Sig. (2-tailed)	0.641	0.622	0.621	0.775	0.160	0.485
Glycine (3.56)	Pearson Correlation	0.195	-0.017	0.020	-0.060	0.000	-0.038
	Sig. (2-tailed)	0.054	0.871	0.848	0.559	0.998	0.712
Creatine (3.95)	Pearson Correlation	0.058	-0.110	-0.077	-0.134	-0.027	-0.129

	Sig. (2-tailed)	0.573	0.281	0.451	0.189	0.789	0.206
Creatine phosphate (3.96)	Pearson Correlation	0.109	-0.067	-0.025	-0.103	0.012	-0.108
	Sig. (2-tailed)	0.287	0.515	0.804	0.314	0.910	0.290
Lactate (4.11)	Pearson Correlation	-0.100	0.118	0.096	0.074	0.042	0.077
	Sig. (2-tailed)	0.327	0.249	0.345	0.472	0.679	0.453
Proline (4.15)	Pearson Correlation	-0.051	-0.168	-0.151	-0.175	0.043	-0.163
	Sig. (2-tailed)	0.617	0.098	0.137	0.085	0.671	0.108
Threonine (4.25)	Pearson Correlation	-0.150	-0.157	-0.138	-0.172	0.042	-0.045
	Sig. (2-tailed)	0.141	0.122	0.175	0.090	0.679	0.663
**Glucose (4.65)**	Pearson Correlation	0.042	**0.239***	**0.207***	0.175	-0.058	0.115
	Sig. (2-tailed)	0.679	**0.018**	**0.041**	0.085	0.573	0.259
Triglycerides (5.08)	Pearson Correlation	-0.129	-0.111	-0.098	-0.025	-0.083	0.135
	Sig. (2-tailed)	0.206	0.276	0.338	0.810	0.417	0.187
Unsaturated fatty acids (5.20)	Pearson Correlation	-0.069	-0.121	-0.108	-0.038	-0.074	0.106
	Sig. (2-tailed)	0.500	0.235	0.291	0.712	0.471	0.297
Tyrosine (6.90)	Pearson Correlation	-0.043	0.013	0.014	-0.030	0.109	0.054
	Sig. (2-tailed)	0.673	0.895	0.889	0.766	0.284	0.597
**Histidine (7.08)**	Pearson Correlation	0.186	-0.183	-0.167	**-0.200***	-0.021	-0.077
	Sig. (2-tailed)	0.067	0.071	0.100	**0.049**	0.836	0.453
**Phenylalanine (7.42)**	Pearson Correlation	**0.245***	0.074	0.092	0.115	0.013	0.110
	Sig. (2-tailed)	**0.015**	0.466	0.366	0.258	0.901	0.282
**Formate (8.45)**	Pearson Correlation	**-0.215***	0.120	0.127	0.123	0.125	0.073
	Sig. (2-tailed)	**0.033**	0.240	0.213	0.228	0.221	0.473

BMI, body mass index; PPD, probing pocket depth; CAL, clinical attachment level; PS, periodontitis stages; BOP, bleeding on probing; * Correlation is significant at the 0.05 level (2-tailed); ** Correlation is significant at the 0.01 level (2-tailed). Metabolite names and values in bold represent statistical difference ($p < 0.05$).

Table S2. Pearson correlation among the salivary metabolites and co-variables related to BMI and periodontal parameters.

Metabolites (chemical shift - ppm)		BMI	PPD	CAL	PS	Dental plaque	BOP
**3-Methyl-2-ketovaleric acid (0.85)**	Pearson Correlation	-0.142	**0.472****	**0.514****	**0.415****	**0.225***	**0.424****
	Sig. (2-tailed)	0.164	**<0.001**	**<0.001**	**<0.001**	**0.026**	**<0.001**
**Unknown 1 (0.87)**	Pearson Correlation	-0.185	**0.591****	**0.614****	**0.490****	**0.284****	**0.522****
	Sig. (2-tailed)	0.068	**<0.001**	**<0.001**	**<0.001**	**0.005**	**<0.001**
**Butyric acid (0.89)**	Pearson Correlation	-0.006	**0.636****	**0.637****	**0.510****	**0.412****	**0.522****
	Sig. (2-tailed)	0.956	**<0.001**	**<0.001**	**<0.001**	**<0.001**	**<0.001**
**Isovaleric acid (0.91)**	Pearson Correlation	-0.083	**0.556****	**0.562****	**0.467****	**0.374****	**0.550****
	Sig. (2-tailed)	0.418	**<0.001**	**<0.001**	**<0.001**	**<0.001**	**<0.001**
**Leucine (0.97)**	Pearson Correlation	-0.055	**0.525****	**0.547****	**0.429****	**0.266****	**0.449****
	Sig. (2-tailed)	0.592	**<0.001**	**<0.001**	**<0.001**	**0.008**	**<0.001**
**Valine (0.99)**	Pearson Correlation	-0.038	**0.525****	**0.531****	**0.425****	**0.262****	**0.463****
	Sig. (2-tailed)	0.708	**<0.001**	**<0.001**	**<0.001**	**0.009**	**<0.001**
**Isoleucine (1.01)**	Pearson Correlation	-0.016	**0.438****	**0.436****	**0.331****	**0.238***	**0.402****
	Sig. (2-tailed)	0.879	**<0.001**	**<0.001**	**0.001**	**0.018**	**<0.001**
**Propionic acid (1.06)**	Pearson Correlation	0.174	**0.409****	**0.387****	**0.320****	**0.454****	**0.402****
	Sig. (2-tailed)	0.086	**<0.001**	**<0.001**	**0.001**	**<0.001**	**<0.001**
**2,3-Butanediol (1.14)**	Pearson Correlation	**0.234***	-0.079	-0.077	-0.043	-0.006	0.012
	Sig. (2-tailed)	**0.020**	0.442	0.453	0.677	0.952	0.904
**Ethanol (1.18)**	Pearson Correlation	0.122	-0.016	-0.019	-0.032	0.023	0.001
	Sig. (2-tailed)	0.231	0.874	0.855	0.752	0.825	0.992
**Fucose (1.25)**	Pearson Correlation	0.007	0.118	0.105	0.013	0.102	0.091
	Sig. (2-tailed)	0.948	0.248	0.305	0.895	0.317	0.371
**Acetoin (1.38)**	Pearson Correlation	0.144	0.099	0.109	0.084	0.064	0.136
	Sig. (2-tailed)	0.157	0.334	0.286	0.413	0.533	0.183
**Alanine (1.48)**	Pearson Correlation	-0.057	**0.292****	**0.330****	0.169	0.133	0.194
	Sig. (2-tailed)	0.579	**0.004**	**0.001**	0.096	0.192	0.055
**Putrescine (1.76)**	Pearson Correlation	0.040	**0.324****	**0.330****	**0.221***	**0.304****	**0.292****
	Sig. (2-tailed)	0.692	**0.001**	**0.001**	**0.029**	**0.002**	**0.004**
**Acetic acid (1.92)**	Pearson Correlation	0.084	**0.302****	**0.281****	**0.228***	**0.329****	**0.332****
	Sig. (2-tailed)	0.411	**0.003**	**0.005**	**0.024**	**0.001**	**0.001**
**N-acetylneuraminic acid (2.21)**	Pearson Correlation	-0.023	**0.327****	**0.332****	**0.224***	**0.254***	**0.276****
	Sig. (2-tailed)	0.824	**0.001**	**0.001**	**0.027**	**0.012**	**0.006**
**5-Aminopentoate (2.24)**	Pearson Correlation	0.078	0.163	0.162	0.064	**0.220***	0.177
	Sig. (2-tailed)	0.446	0.108	0.112	0.530	**0.029**	0.081
**N-acetylglutamine (2.33)**	Pearson Correlation	-0.081	-0.004	0.056	-0.072	0.040	-0.067
	Sig. (2-tailed)	0.428	0.972	0.583	0.484	0.693	0.509
**Pyruvic acid (2.37)**	Pearson Correlation	0.049	<0.001	0.044	-0.066	-0.044	-0.074
	Sig. (2-tailed)	0.634	0.998	0.665	0.518	0.667	0.469
**Succinic acid (2.41)**	Pearson Correlation	**0.245***	0.092	0.129	0.069	0.026	0.050
	Sig. (2-tailed)	**0.015**	0.369	0.205	0.500	0.796	0.628
**Unknown 2 (2.49)**	Pearson Correlation	-0.117	**0.442****	**0.474****	**0.328****	**0.231***	**0.373****
	Sig. (2-tailed)	0.249	**<0.001**	**<0.001**	**0.001**	**0.022**	**<0.001**
**Citric acid (2.53)**	Pearson Correlation	-0.025	-0.031	0.038	-0.018	-0.021	-0.022
	Sig. (2-tailed)	0.807	0.761	0.709	0.863	0.838	0.830
**Methylamine (2.60)**	Pearson Correlation	-0.141	**0.291****	**0.303****	0.189	0.122	**0.290****
	Sig. (2-tailed)	0.165	**0.004**	**0.002**	0.062	0.232	**0.004**
**Trimethylamine (2.89)**	Pearson Correlation	-0.113	**0.555****	**0.556****	**0.455****	**0.298****	**0.568****

	Sig. (2-tailed)	0.269	**<0.001**	**<0.001**	**<0.001**	**0.003**	**<0.001**
**Ornithine (3.06)**	Pearson Correlation	0.050	**0.345****	**0.353****	**0.254***	**0.289****	**0.301****
	Sig. (2-tailed)	0.627	**0.001**	**<0.001**	**0.012**	**0.004**	**0.003**
**Choline (3.20)**	Pearson Correlation	-0.143	**0.246***	**0.279****	**0.206***	0.036	**0.277****
	Sig. (2-tailed)	0.159	**0.015**	**0.005**	**0.042**	0.722	**0.006**
**Citrulline (3.14)**	Pearson Correlation	-0.058	0.107	0.101	0.031	0.032	0.116
	Sig. (2-tailed)	0.570	0.293	0.322	0.762	0.755	0.257
**Dimethylsulfone (3.15)**	Pearson Correlation	-0.024	-0.044	-0.045	-0.008	-0.059	0.017
	Sig. (2-tailed)	0.817	0.666	0.661	0.941	0.562	0.869
**Methanol (3.36)**	Pearson Correlation	-0.084	-0.082	-0.082	-0.100	-0.057	-0.125
	Sig. (2-tailed)	0.410	0.421	0.422	0.327	0.578	0.220
**Taurine (3.43)**	Pearson Correlation	0.083	**0.274****	**0.289****	0.181	0.075	**0.279****
	Sig. (2-tailed)	0.415	**0.006**	**0.004**	0.075	0.463	**0.005**
**Glycine (3.56)**	Pearson Correlation	**0.246***	-0.065	-0.026	-0.074	0.108	0.028
	Sig. (2-tailed)	**0.015**	0.523	0.797	0.471	0.290	0.787
**Lactate (4.12)**	Pearson Correlation	**0.225***	-0.010	0.003	-0.059	-0.111	0.014
	Sig. (2-tailed)	**0.026**	0.923	0.974	0.564	0.278	0.892
**Proline (4.14)**	Pearson Correlation	-0.038	-0.070	-0.053	-0.142	0.020	-0.080
	Sig. (2-tailed)	0.708	0.496	0.601	0.163	0.843	0.434
**Glucose (5.24)**	Pearson Correlation	0.183	-0.021	0.008	-0.047	-0.028	0.074
	Sig. (2-tailed)	0.071	0.836	0.941	0.646	0.786	0.470
**Unknown sugar (5.27)**	Pearson Correlation	0.044	-0.131	-0.144	**-0.250***	-0.008	-0.082
	Sig. (2-tailed)	0.666	0.198	0.157	**0.013**	0.938	0.420
**Maltose (5.41)**	Pearson Correlation	**0.215***	-0.098	-0.061	-0.067	0.025	0.042
	Sig. (2-tailed)	**0.033**	0.339	0.549	0.512	0.811	0.680
**Urea (5.78)**	Pearson Correlation	-0.055	-0.162	-0.145	-0.075	**-0.351****	-0.160
	Sig. (2-tailed)	0.593	0.111	0.154	0.465	**<0.001**	0.115
**Uridine (5.91)**	Pearson Correlation	-0.085	-0.131	-0.119	-0.129	**-0.397****	-0.126
	Sig. (2-tailed)	0.404	0.200	0.243	0.205	**<0.001**	0.216
**Fumaric acid (6.52)**	Pearson Correlation	0.110	0.017	0.050	-0.020	-0.099	-0.002
	Sig. (2-tailed)	0.279	0.870	0.628	0.844	0.333	0.982
**Unknown 3 (6.84)**	Pearson Correlation	-0.055	**0.292****	**0.317****	**0.256***	0.187	**0.223***
	Sig. (2-tailed)	0.588	**0.004**	**0.001**	**0.011**	0.065	**0.028**
**para-Hydroxyphenylacetic acid (6.87)**	Pearson Correlation	0.059	0.130	0.163	0.111	0.203*	0.080
	Sig. (2-tailed)	0.567	0.203	0.108	0.277	0.045	0.433
**Tyrosine (6.90)**	Pearson Correlation	0.047	0.155	0.212*	0.131	0.031	0.030
	Sig. (2-tailed)	0.643	0.128	0.036	0.200	0.765	0.771
**Histidine (7.07)**	Pearson Correlation	0.022	0.133	0.181	0.124	0.028	0.021
	Sig. (2-tailed)	0.831	0.191	0.074	0.223	0.785	0.836
**Phenylalanine (7.43)**	Pearson Correlation	0.039	**0.389****	**0.388****	**0.300****	0.189	**0.307****
	Sig. (2-tailed)	0.706	**<0.001**	**<0.001**	**0.003**	0.063	**0.002**
**Unknown 4 (8.19)**	Pearson Correlation	0.015	**0.287****	**0.305****	0.190	-0.113	0.141
	Sig. (2-tailed)	0.882	**0.004**	**0.002**	0.061	0.268	0.165
**Unknown 5 (8.21)**	Pearson Correlation	-0.031	**0.266****	**0.292****	0.166	-0.097	0.125
	Sig. (2-tailed)	0.760	**0.008**	**0.004**	0.102	0.340	0.218
**Formic acid (8.45)**	Pearson Correlation	0.133	0.071	0.059	0.152	0.080	0.010
	Sig. (2-tailed)	0.190	0.486	0.567	0.136	0.435	0.925

BMI, body mass index; PPD, probing pocket depth; CAL, clinical attachment level; PS, periodontitis stages; BOP, bleeding on probing; * Correlation is significant at the 0.05 level (2-tailed); ** Correlation is significant at the 0.01 level (2-tailed). Metabolite names and values in bold represent statistical difference ($p < 0.05$).
